# Development of a Larval Zebrafish Model for Acute Organophosphorus Nerve Agent and Pesticide Exposure and Therapeutic Evaluation

**DOI:** 10.3390/toxics8040106

**Published:** 2020-11-17

**Authors:** Jeffrey A. Koenig, Cindy Acon Chen, Tsung-Ming Shih

**Affiliations:** Medical Toxicology Research Division, Army Medical Research Institute of Chemical Defense, Aberdeen Proving Ground, MD 21010-5400, USA; jkoenig@som.umaryland.edu (J.A.K.); cindy.acon-chen.civ@mail.mil (C.A.C.)

**Keywords:** acetylcholinesterase, cholinesterase inhibitor, organophosphorus compound, oxime reactivator, zebrafish

## Abstract

Organophosphorus compound exposure remains a present threat through agricultural accidents, warfare, or terrorist activity. The primary mechanism of organophosphorus toxicity is through inhibition of the enzyme acetylcholinesterase, with current emergency treatment including anticholinergics, benzodiazepines, and oxime reactivators. However, a need for more effective and broadly acting countermeasures remains. This study aimed to develop larval zebrafish as a high-throughput model for evaluating novel therapeutics against acute organophosphorus exposure. Larval zebrafish at six days post-fertilization were exposed to acute concentrations of seven organophosphorus compounds and treated with one of three oximes. Lethality studies indicated similar relative toxicity to that seen in the established rodent model, with chemical warfare agents proving more lethal than organophosphorus pesticides. Additionally, the organophosphorus-specific response for oxime reactivation of acetylcholinesterase was comparable to what has been previously reported. Behavioral studies measuring the visual motor response demonstrated greater efficacy for centrally acting oxime compounds than for those that are contained to the peripheral tissue. Overall, these results support the use of this larval zebrafish model as a high-throughput screening platform for evaluating novel treatments following acute organophosphorus exposure.

## 1. Introduction

Organophosphorus (OP) compounds, to include agricultural pesticides and chemical warfare nerve agents (CWNA), are highly toxic substances, acute exposure to which remains a present threat via occupational contact, terrorism-related release, or suicidal ingestion. The primary mechanism of action of these compounds is through potent inhibition of the enzyme acetylcholinesterase (AChE) [1,2]. Inhibition of this enzyme leads to an accumulation of the neurotransmitter acetylcholine at central and peripheral cholinergic synapses. The overstimulation of the cholinergic pathways has numerous toxic consequences that are dependent on the severity of the OP exposure. These acute symptoms include miosis, hypersecretions, gastrointestinal and respiratory distress, convulsions, and prolonged seizure activity (status epilepticus). OP-induced lethality is primarily attributed to respiratory paralysis originating both centrally and peripherally [1,2,3].

The primary medical intervention against OP exposure is prompt administration of an anticholinergic drug, specifically atropine sulfate. This compound antagonizes the activity of muscarinic acetylcholine receptors, reducing the ongoing overstimulation. An oxime AChE reactivator such as pralidoxime chloride (2-PAM) may also be administered concomitantly to reactivate any unaged AChE [4]. Additionally, benzodiazepines (e.g., diazepam or midazolam) may be utilized to control ongoing seizure activity initiated within the central nervous system (CNS) [5,6]. There are limitations on the current treatment regimens, however. For example, the commonly approved oximes, such as 2-PAM or HI-6, possess quaternary nitrogen structures that preclude their crossing of the blood-brain barrier (BBB), limiting their efficacy only to the periphery [7,8].

The typical research progression for novel OP exposure therapeutics begins with either in silico or in vitro modeling, with those compounds demonstrating initial efficacy moved into the well-established in vivo rodent model [9]. While this has been an effective model, it does not lend itself well to the increasing need for high-throughput drug discovery. In recent years, the zebrafish (*Danio rerio*) has been utilized as an alternative vertebrate model in both toxicology and drug discovery research [10,11,12,13]. Zebrafish possess a well-conserved physiology that can be directly visualized in the transparent embryos and larvae [14,15]. They additionally lend themselves well to behavioral studies, which can be performed in an automated, high-throughput manner [16]. Their specific advantages as a model for OP exposure include a well conserved AChE amino acid sequence and a complete absence of butyrylcholinesterase (BChE) expression, which could confound AChE inhibition and reactivation studies [17,18]. Additionally, the conserved trends of OP inhibition and oxime reactivation in vitro between zebrafish and human AChE have been reported [19].

When modeling OP exposure, the zebrafish has primarily been used for chronic or low dose exposure paradigms [10,20]. While this can offer insight into some of the mechanisms underlying OP toxicity, novel treatment options for an acute, high-dose exposure will need to uniquely and specifically address the immediate life-threatening symptoms mentioned previously [21,22]. Therefore, this study aimed to develop a larval zebrafish model of acute OP exposure. This included establishing the median lethal concentration (LC_50_) of seven OP compounds and the AChE-inhibiting capabilities of each. These OP compounds, including three CWNAs, are sarin (GB), soman (GD), cyclosarin (GF), diisopropylfluorophosphate (DFP), chlorpyrifos (CP), chlorpyrifos oxon (CPO), and paraoxon (PO). The three oxime compounds evaluated for their ability to restore AChE activity were 2-PAM, MMB-4, and MINA. The compound 2-PAM is the emergency OP antidote currently approved by the United States; however, MMB-4 is under investigation due to its reported broader efficacy [23]. MINA has recently received renewed interest because, unlike 2-PAM and MMB-4, it possesses an uncharged nitrogen that allows for a greater propensity to cross the BBB [24,25]. The efficacy of these oxime compounds was determined both behaviorally and through direct AChE activity assays. Our initial results support the use of this model as a high-throughput screening system for novel treatments of OP exposure.

## 2. Materials and Methods

### 2.1. Husbandry and Larvae Production

Adult zebrafish of the wild-type AB strain were maintained under standard conditions within a recirculating system on a 14 h light: 10 h dark photoperiod at 28 °C. Embryos were collected utilizing a static tank strategy and screened for viability under a dissecting microscope before being transferred to a 100 mm petri dish containing E3 embryo medium (5.0 mM NaCl, 0.17 mM KCl, 0.33 mM CaCl, 0.33 mM MgSO_4_) and methylene blue (100 μL/L of 0.1% stock solution). Larvae and embryos were maintained on the same 14 h light: 10 h dark photoperiod at 28 °C under fasting conditions throughout the entire experimental period. All procedures were approved by the Institutional Animal Care and Use Committee (IACUC) of USAMRICD, an AAALAC accredited organization, on 3 March 2016.

### 2.2. Chemicals

Sodium chloride, potassium chloride, calcium chloride, magnesium sulfate, chlorpyrifos (CP), paraoxon-ethyl (PO), DFP (diisopropylfluorophosphate), MINA (monoisonitrosoacetone), methylene blue, and acetylthiocholine iodide were acquired from Sigma-Aldrich (St. Louis, MO, USA). The 2-PAM (pralidoxime chloride) was acquired from Baxter Healthcare (Deerfield, IL, USA). The bicinchoninic acid (BCA) protein assay and DTNB (5,5′-dithiobis-(2-nitrobenzoic acid); Ellman’s reagent) were acquired from Thermo Fisher Scientific (Waltham, MA, USA). Chlorpyrifos oxon (CPO) was acquired from Chem Service (West Chester, PA, USA). Sarin (GB; isopropyl methylphosphonofluoridate), soman (GD; O-pinacolyl methylphosphonofluoridate), and cyclosarin (GF; cyclohexyl methylphosphonofluoridate) were obtained from the U.S. Army Combat Capabilities Development Command Chemical Biological Center (Aberdeen Proving Ground, MD, USA). MMB-4 (1,1′-methylenebis[4[(hydroxyimino)methyl]-pyridinium]dimethanesulfonate) was acquired from the Division of Experimental Therapeutics, Walter Reed Army Institute of Research (Silver Spring, MD, USA).

### 2.3. Exposure and Treatment Paradigm

All experimental procedures were performed with 6 days post-fertilization (dpf) zebrafish larvae of the AB strain. Exposures and treatments were conducted in 12-well plates with Netwell inserts (Corning Inc.) to allow for the efficient transfer of larvae from plate to plate. Each well contained approximately 4.0 mL of solution, and all experimental solutions were diluted in E3 medium. The exposure and treatment paradigm consisted of transferring zebrafish larvae (20 per well) from a plate containing E3 medium onto a plate containing the diluted OP compound. The larvae remained in the OP solution for the experimentally dictated time before being transferred to two successive wash plates containing fresh E3 medium for 5 min each. Larvae were then transferred to a plate containing the oxime solutions and treated for 20 min. A final 5-min wash was conducted before the larvae were either transferred to individual wells of a 96-well plate (lethality and behavioral experiments) or humanely euthanized and placed in 1.5 mL conical tubes (AChE activity determination). All exposure and treatment experiments were performed in triplicate. Collected samples were frozen and stored at −80 °C for later analysis.

### 2.4. AChE Activity Assay

AChE activity was determined utilizing a modified Ellman et al. [26] microplate assay similar to what has been described previously [23]. Twenty larvae were pooled within each sample and homogenized in a 1% Triton-X 100 solution utilizing a motorized pestle (Power Masher II). Samples were centrifuged at 21,000× *g* for 10 min at 4 °C, and supernatant was collected. On a 96-well plate, 7 µL of sample, 20 µL of deionized water, and 200 µL of DTNB (0.424 mM in 50 mM Tris buffer; pH 8.2) were added to each well. The plate was allowed to incubate/shake at 37 °C and 400 rpm for 10 min. Acetylthiocholine iodide (30 µL; 51.4 mM) was added to each well, and kinetic absorbance was determined at 412 nm. Total protein concentration for each sample was determined utilizing the BCA protein assay. The AChE activity was then expressed as the µmol of substrate hydrolyzed/min/mg of protein.

### 2.5. Behavioral Assay

Locomotor activity was determined at 4 h and 24 h following the exposure period utilizing a DanioVision system and EthioVision XT software (Noldus Information Technology, Leesburg, VA, USA) as previously described by Faria et al. [27]. In short, larvae were placed in individual wells of a 96-well plate immediately following exposure and treatment. The larvae acclimated within the instrument in the dark at 28 °C for 1 h prior to the recording period. Recordings were made at 4 h and 24 h following treatment. These time points were selected to both capture the immediate efficacy offered by oxime treatment and the innate recovery that is sometimes seen at 24 h (personal observation). The 50 min measurement period was composed of a 20 min dark–10 min light–20 min dark interval to evaluate the visual motor response (VMR). VMR is a robust increase in movement seen in zebrafish larvae during a rapid transition from light to dark. The total distance traveled (mm) was measured in 2 min bins.

### 2.6. Data Analysis

For LC_50_ determination, a probit regression analysis was performed using IBM SPSS Statistics v22. An exploratory analysis was conducted to compare the OP compound LC_50_s at four exposure times (30, 60, 90, and 120 min) using an analysis of variance (ANOVA) with the compound and time as factors. Since compounds CP and CPO had no LC_50_ estimates at 30 min and 120 min, respectively, a regression analysis using the least squares methods, which adjusted the means with respect to the missing LC_50_s, was performed including all compounds and times available. If significant compound and/or time effects were observed, a Tukey’s multiple comparison test was used to compare all pairs of compounds or pairs of times. For mean comparisons in the oxime AChE reactivation and behavioral studies, an ANOVA with a Dunnett’s multiple comparison test was utilized. Results were considered significant if *p* < 0.05.

## 3. Results

### 3.1. OP Lethality and AChE Inhibition

**Figure 1:**
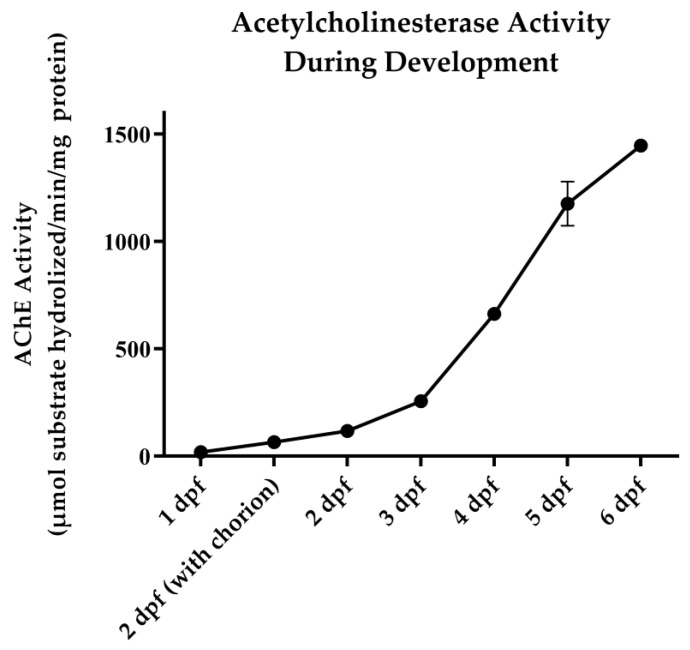
Developmental AChE activity. Zebrafish larvae were collected every 24 h from 1 day post fertilization (dpf) to 6 dpf, and AChE activity was determined. Data represent the average ± SEM, with 3 samples (20 pooled larvae) per group.

Baseline AChE activity was initially evaluated by a time-course collection of zebrafish embryos and larvae every 24 h, 1 dpf through 6 dpf. There was a steady increase in AChE activity from 18.9 to 1448.0 µmol of substrate hydrolyzed/min/mg of protein on 1 dpf and 6 dpf, respectively (Figure 1). An additional collection was made at 2 dpf for embryos that remained in their chorion at this time point, with greater AChE activity seen in hatched larvae. Six dpf larvae were utilized for all subsequent experiments.

To develop our model of larval zebrafish OP exposure, the LC_50_ concentration had to first be determined for each of the seven OP compounds. They were GB, GD, GF, DFP, CP, CPO, and PO. Six dpf zebrafish were exposed to varying concentrations of each OP compound (GB, GD, GF, PO, CPO: 0.064–1000 µM; DFP, CP: 0.64 µM–25 mM) for 30, 60, 90, or 120 min. Lethality was recorded 24 h after exposure as determined by cessation of heartbeat, with the LC_50_ values listed in Table 1. The CWNAs were generally more toxic than the OP pesticide compounds, with CP demonstrating the least toxicity. CPO, the active metabolite of CP, was significantly more toxic than the parent compound. The overall rank order of toxicity was GD > GB = GF = CPO > DFP = PO > CP. Duration of exposure had a significant effect across all compounds. Effects on gross morphology were in line with what has been described previously [27]. The most commonly noted with moderate exposure across all OP compounds were muscle paralysis and lowered heart rate. Severe exposures additionally produced shortened trunk length and an enlarged swim bladder (data not shown).

**Figure 2:**
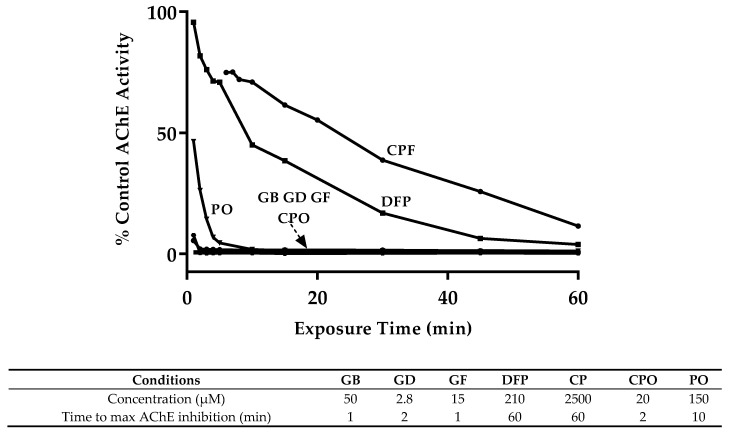
Time-course inhibition of AChE activity. Zebrafish larvae (6 dpf) were exposed to the listed OP compound and concentration from 1 to 60 min before collection to determine the degree of AChE inhibition. Data represent the average AChE activity when compared to non-exposed control, with 3 samples (20 pooled larvae) per group per time point.

To determine the time-course of AChE inhibition, we exposed 6 dpf larvae to the lower end of the 95% confidence interval for the 60 min LC_50_ concentration with exposure times ranging from 1–60 min (Figure 2). This concentration was selected to minimize overall lethality while still maintaining OP specificity. GB, GD, GF, and CPO quickly reached maximum inhibition with <2% AChE activity remaining within 1–2 min, while PO exposure required 10 min. CP and DFP were unable to reach maximum inhibition within the experimental window, producing AChE activity of 11% and 4% of baseline, respectively, at 60 min.

### 3.2. Oxime Reactivation

Utilizing the exposure times and concentrations determined in the previous experiments, we next evaluated the ability of three oxime compounds (2-PAM, MMB-4, and MINA) to reactivate AChE inhibited by GB, GD, GF, CPO, or PO. DFP and CP were excluded from the study due to their relatively slower rate of inhibition. Oxime treatment was delivered at either 200 µM or 400 µM for 20 min.

**Figure 3:**
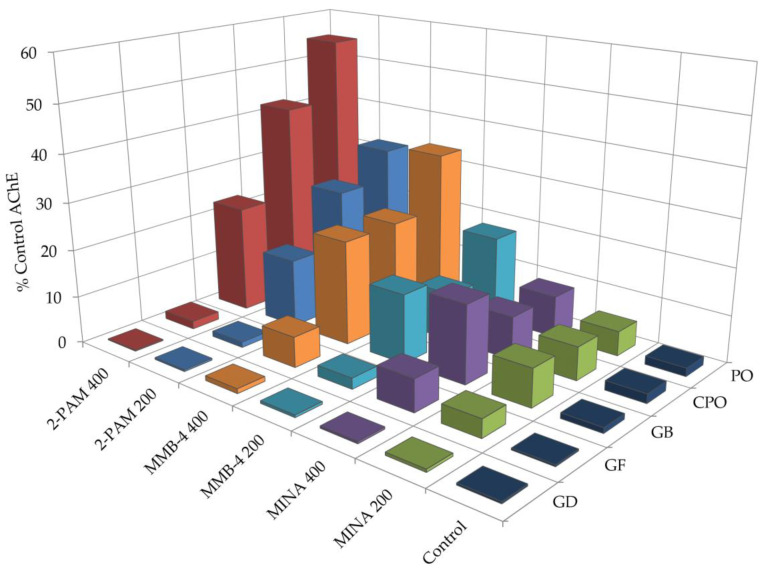
Oxime reactivation of OP-inhibited AChE activity. Following OP exposure, zebrafish larvae (6 dpf) were treated with 2-PAM, MMB-4, or MINA at 200 µM or 400 µM for 20 min. Data represent the percentage of AChE activity compared to non-exposed vehicle control, with 3–6 samples (20 pooled larvae) per group.

The AChE reactivation results are summarized in Figure 3 and Table S1. Overall, CPO and PO proved more responsive to oxime treatment when compared to the CWNAs (i.e., GB, GD, and GF). All oxime treatments significantly reactivated CPO-inhibited AChE, with 2-PAM (400 µM) proving the most efficacious, restoring activity to 42.0% of baseline. A similar trend was seen in PO-inhibited AChE, with 2-PAM restoring AChE activity to 54.9%. MINA reactivation did not reach significant levels. All three oximes were significantly efficacious following GB exposure with 2-PAM (400 µM) and MMB-4 (400 µM), restoring AChE activity to 22.8% and 22.1%, respectively. For GF, only MMB-4 (400 µM) and MINA (400 µM) reached significance, restoring AChE activity to 6.5% and 6.8%, respectively. GD-inhibited AChE proved refractory to all oxime treatments.

### 3.3. Behavioral Monitoring

**Figure 4:**
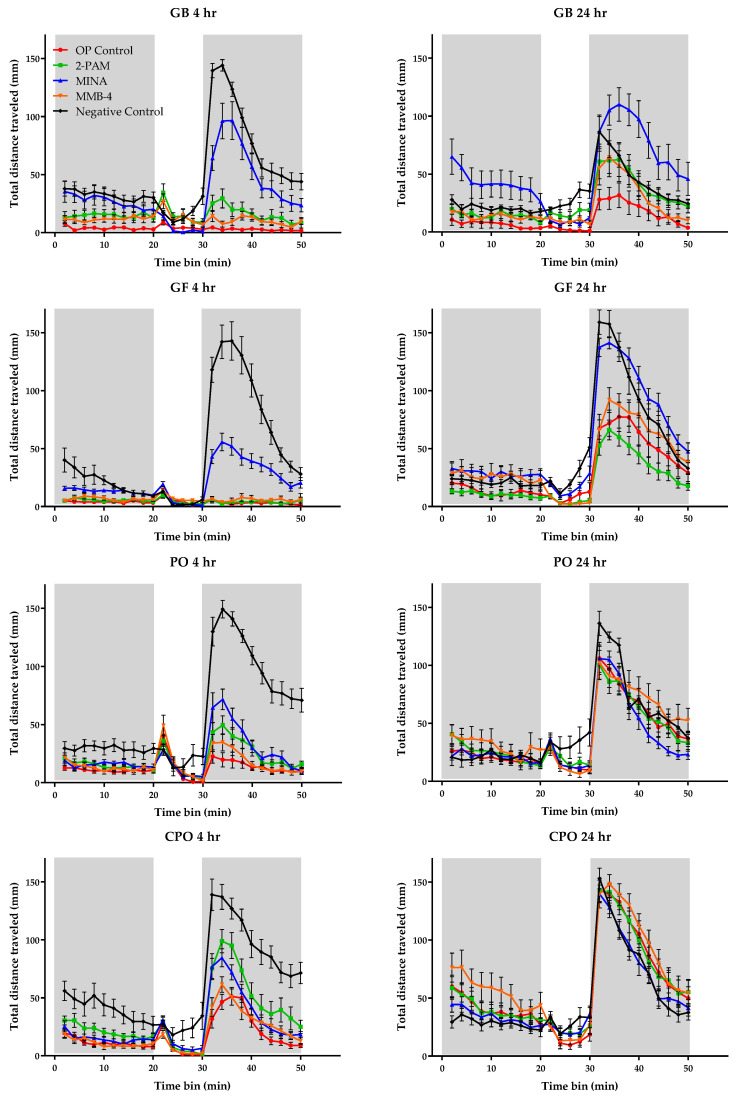
Monitoring of VMR behavior following OP exposure and oxime treatment. Zebrafish larvae (6 dpf) were treated with 2-PAM, MINA, or MMB-4 at 400 µM following OP exposure. VMR was recorded at both 4 and 24 h following treatment. Shaded areas (0–20 min and 30–50 min) represent periods of dark, while the non-shaded areas (20–30 min) represent periods of light. Data points represent the average total distance traveled (mm) ± SEM within a 2 min bin, with 16–34 subjects per group.

To correlate AChE inhibition and reactivation with behavior, VMR was recorded in zebrafish larvae following OP exposure and oxime treatment (at 400 µM). The results of VMR behavior following OP exposure and oxime treatment are shown in Figure 4. There was a general decrease in baseline activity across all OP-exposed groups. VMR was totally ablated in GB-exposed and GF-exposed larvae and partially ablated for PO and CPO exposure. MINA treatment proved the most efficacious overall across the four OP compounds, significantly restoring the VMR when compared to the untreated control for each OP at the 4-h measurement. Additionally, 2-PAM was effective in significantly restoring VMR in the CPO-exposed group. At the 24-h recording, all treatments returned to baseline activity for the CPO and PO exposure groups. Only the MINA-treated group returned to baseline VMR activity following GB or GF exposure.

## 4. Discussion

The goal of the present study was to offer evidence for the feasibility of utilizing zebrafish larvae as a model for acute OP exposure and the evaluation of potential countermeasures. We evaluated the toxicity and AChE-inhibiting properties of seven OP compounds to include 3 CWNAs, and this study was the first of its kind to evaluate CWNA exposure in a zebrafish model. There was an overall agreement in both relative OP lethality and specificity of oxime reactivation of OP-inhibited AChE when compared to the established rodent model [23,28]. The OP pesticide compounds (CPO and PO) proved more responsive to oxime reactivation when compared to the CWNAs (GB, GD, and GF). Additionally, GD proved completely refractory to oxime reactivation, as had been reported in rodent models. This agrees with the well documented ability of the GD-AChE complex to quickly “age”, a spontaneous dealkylation that results in an irreversibly inhibited AChE [29].

One result of note is the seemingly different outcomes seen in the AChE reactivation and VMR behavioral experiments. While in the whole-body AChE reactivation measurements 2-PAM and MMB-4 proved the most efficacious, MINA was shown to provide the greatest efficacy at restoring VMR. One possible explanation could be differing reactivation centrally versus peripherally. MINA possesses an uncharged tertiary nitrogen structure, and while this lowers its affinity for the OP-AChE complex when compared to 2-PAM and MMB-4, it greatly increases its ability to cross the BBB [24,25], with the zebrafish BBB being established by 3 dpf [30]. It is possible that the increased AChE reactivation in the brain better reestablishes neurotransmitter homeostasis in the environment containing the intrinsically photoreceptive cells driving VMR [31]. This is supported by recent research describing the synergistic relationship between melanopsin-dependent phototransduction in intrinsically photosensitive retinal ganglion cells (ipRGCs) and cholinergic pathways [32,33]. This observation could be advantageous in future screening to discriminate those compounds that are capable of crossing the BBB, an attractive characteristic in potential therapeutics. Additional work would be needed to further investigate the physiological and molecular mechanisms driving the VMR disturbance in our model. Previous research has demonstrated multiple pathologies affecting the visual system following OP exposure which included neural necrosis, upsetting of the retinal architecture, and down-regulation of phototransduction-related pathways [27].

Overall, this study provides support for the use of the larval zebrafish as a high-throughput alternative in vivo model for acute OP exposure and the evaluation of novel treatment compounds, especially the AChE reactivators. Additional work must be done to further refine the model for the screening of large compound libraries. This would include transferring the exposure and treatment procedures from a 12-well to 96-well format and solvent standardization, as many potential small molecule therapies will be lipid soluble. Established biochemical assays and high-throughput imaging techniques could be incorporated as additional endpoints to further evaluate novel compounds [27,34], clarifying potential mechanisms of action, which is one of the primary challenges in high-throughput library screening. The model could also be adjusted to investigate time points beyond what has been established here. By exposing larvae to a single acute OP challenge, it would be possible to monitor the physiological and behavioral detriments that might arise over time following treatment and recovery. This could potentially identify novel mechanisms of injury that differ from the traditional chronic exposure models. Finally, zebrafish amenability to genetic manipulation could provide the opportunity for a human-AChE-expressing zebrafish strain, improving the model’s clinical relevance when screening compounds that potentially interact with the OP-AChE complex.

## Figures and Tables

**Table 1:** Median lethal concentration (LC_50_) determination of organophosphorus compounds in 6 dpf zebrafish larvae.

Compound	Exposure Time (min)				
	30	60	90	120	sig. *
GB	229.8 (151.7–378.1)	61.5 (45.9–83.2)	18.9 (13.6–26.7)	6.9 (4.7–10.0)	c
GD	21.8 (13.1–38.8)	4.0 (2.8–5.5)	2.3 (1.1–4.2)	1.3 (0.7–2.3)	d
GF	30.6 (19.4–50.8)	21.1 (14.0–32.8)	15.4 (10.8–22.3)	6.6 (3.9–12.0)	c
DFP	2805.6 (2271.9–3420.8)	257.4 (207.0–311.1)	227.2 (171.1–307.9)	62.7 (52.3–78.2)	b
CP	ND	3206.4 (2478.0–4177.4)	1191.8 (936.6–1508.2)	931.0 (753.3–1144.9)	a
CPO	85.4 (68.0–107.7)	22.3 (17.5–28.5)	7.7 (5.8–10.4)	ND	c
PO	451.9 (361.9–566.2)	165.8 (134.2–202.3)	82.9 (63.2–109.2)	108.7 (86.4–137.2)	b

Concentrations are expressed in µM (95% CI). ND = not determined. * a–d: Compounds with different letters have significantly different mean LC_50_ values across all exposure times, *p* < 0.05. GB: sarin; GD: soman; GF: cyclosarin; DFP: diisopropylfluorophosphate; CP: chlorpyrifos; CPO: chlorpyrifos oxon; PO: paraoxon.

## Supplementary Materials

The following are available online at https://www.mdpi.com/2305-6304/8/4/106/s1, Table S1: Oxime reactivation of OP-inhibited AChE.

## Disclaimer

The experimental protocol was approved by the Institutional Animal Care and Use Committee (IACUC) at USAMRICD, and all procedures were conducted in accordance with the principles stated in the Guide for the Care and Use of Laboratory Animals, the Public Health Service Policy on Humane Care and Use of Laboratory Animals and the Animal Welfare Act of 1966 (P.L. 89-544), as amended. The views expressed are solely those of the authors and do not necessarily represent the official views of the CCRP, NIAID, NIH, HHS, USAMRICD or DoD.
