# Supplementary material for: Development of a Larval Zebrafish Model for Acute Organophosphorus Nerve Agent and Pesticide Exposure and Therapeutic Evaluation
Source: Toxics. 2020 Nov 17;8(4):106. doi: 10.3390/toxics8040106 (PMC7712847; doi:10.3390/toxics8040106)
Supplement: Supplementary file 1 [file toxics-08-00106-s001.docx]

Supplementary Materials: Development of a Larval Zebrafish Model for Acute Organophosphorus Nerve Agent and Pesticide Exposure and Therapeutic Evaluation

Jeffrey A. Koenig, Cindy Acon Chen and Tsung-Ming Shih

**Table S1.** Oxime reactivation of OP-inhibited AChE.

| **Average % Control AChE Activity (± SEM)** | | | | | | | |
| --- | --- | --- | --- | --- | --- | --- | --- |
| **Compound** | **Vehicle-**  **Treated** | **2-PAM** | | **MMB-4** | | **MINA** | |
|  |  | **200 µM** | **400 µM** | **200 µM** | **400 µM** | **200 µM** | **400 µM** |
| GB | 1.14 ± 0.05 | 14.53 ± 1.19 | 22.80 ± 1.34 | 14.63 ± 2.27 | 22.12 ± 2.36 | 8.04 ± 0.11 | 16.56 ± 1.00 |
| GD | 0.38 ± 0.04 | 0.39 ± 0.12 | 0.23 ± 0.01 | 0.52 ± 0.02 | 0.99 ± 0.20 | 0.59 ± 0.05 | 0.44 ± 0.07 |
| GF | 0.34 ± 0.03 | 1.16 ± 0.04 | 1.67 ± 0.04 | 2.30 ± 0.23 | 6.47 ± 1.06 | 3.84 ± 0.15 | 6.82 ± 0.64 |
| PO | 1.77 ± 0.15 | 32.27 ± 1.37 | 54.89 ± 6.01 | 18.19 ± 2.84 | 33.75 ± 5.75 | 5.26 ± 0.32 | 8.84 ± 0.17 |
| CPO | 1.96 ± 0.07 | 25.96 ± 2.94 | 42.05 ± 1.65 | 10.13 ± 1.80 | 22.18 ± 4.10 | 7.07 ± 1.27 | 9.15 ± 0.33 |

Shaded boxes = *p* < 0.05 vs. vehicle-treated.
